# Cognitive functioning as a predictor of employment status in relapsing-remitting multiple sclerosis: a 2-year longitudinal study

**DOI:** 10.1007/s10072-019-03999-w

**Published:** 2019-07-19

**Authors:** Dennis A.M. van Gorp, Karin van der Hiele, Marco A.P. Heerings, Peter J. Jongen, Jac J.L. van der Klink, Michiel F. Reneman, Edo P.J. Arnoldus, Ernesto A.C. Beenakker, Jeroen J.J. van Eijk, Stephan T.F.M. Frequin, Koen de Gans, Elske Hoitsma, Jop P. Mostert, Wim I.M. Verhagen, Désirée Zemel, Leo H. Visser, Huub A.M. Middelkoop

**Affiliations:** 1 National Multiple Sclerosis Foundation, Mathenesserlaan 378, 3023 HB Rotterdam, The Netherlands; 2 Department of Psychology, Health, Medical and Neuropsychology Unit, Leiden University, PO Box 9555, 2300 RB Leiden, The Netherlands; 3 Department of Neurology, Elisabeth-TweeSteden Hospital, PO Box 90151, 5000 LC Tilburg, The Netherlands; 4 Department of Care Ethics, University of Humanistic Studies, PO Box 797, 3500 AT Utrecht, Utrecht The Netherlands; 5 Department of Health Sciences, Community and Occupational Medicine, University Medical Centre Groningen, PO Box 30001, 9700 RB Groningen, The Netherlands; 6 MS4 Research Institute, Ubbergseweg 34, 9522 KJ Nijmegen,, The Netherlands; 7 Department of Social and Behavioral Sciences, Tranzo Scientific Centre for Care and Welfare, Tilburg University, PO Box 90153, 5000 LE Tilburg, The Netherlands; 8 Department of Rehabilitation Medicine, Center for Rehabilitation, University of Groningen, University Medical Center Groningen, PO Box 30.002, 9750 RA Haren, The Netherlands; 9 Department of Neurology, Medical Centre Leeuwarden, PO Box 888, 8901 BR Leeuwarden, The Netherlands; 10 Department of Neurology, Jeroen Bosch Hospital, PO Box 90153, 2000 ME ’s-Hertogenbosch, The Netherlands; 11 Department of Neurology, St. Antonius Hospital, PO Box 2500, 3430 EM Nieuwegein, The Netherlands; 12 Department of Neurology, Groene Hart Hospital, PO Box 1098, 2800 BB Gouda, The Netherlands; 13 Department of Neurology, Alrijne Hospital Leiden, PO Box 9650, 2300 RD Leiden, The Netherlands; 14 Department of Neurology, Rijnstate Hospital, PO Box 9555, 6800 TA Arnhem, The Netherlands; 15 Department of Neurology, Canisius-Wilhelmina Hospital, PO Box 9015, 6500 GS Nijmegen, The Netherlands; 16 Department of Neurology, Albert Schweitzer Hospital, PO Box 444, 330 AK Dordrecht, The Netherlands; 17 Department of Neurology, Leiden University Medical Centre, PO Box 9600, 2300 RC Leiden, The Netherlands

**Keywords:** Multiple sclerosis, Work, Employment, Cognition, Executive function, Physically disabled

## Abstract

**Background:**

Cognitive functioning has been linked to employment outcomes in multiple sclerosis (MS) in cross-sectional studies. Longitudinal studies are however lacking and previous studies did not extensively examine executive functioning.

**Objectives:**

We examined whether baseline cognitive functioning predicts a change in employment status after 2 years, while taking into account mood, fatigue and disability level.

**Methods:**

A total of 124 patients with relapsing-remitting MS (pwMS) and 60 healthy controls were included. They underwent neurological and neuropsychological examinations and completed online questionnaires. PwMS were divided into a stable and deteriorated employment status group (SES and DES), based on employment status 2 years after baseline. We first examined baseline differences between the SES and DES groups in cognitive functioning, mood, fatigue and disability level. A logistic regression analysis was performed, with change in employment status (SES/DES) as dependent variable.

**Results:**

The DES group included 22% pwMS. Group differences were found in complex attention, executive functioning, self-reported cognitive functioning, fatigue and physical disability. More physical disability (OR = 1.90, *p* = 0.01) and lower executive functioning (OR = 0.30, *p* = 0.03) were retained as independent predictors of DES (*R*^2^ = 0.22, *p* ≤ 0.001).

**Conclusions:**

Baseline physical disability and executive functioning, but none of the other variables, moderately predicted a deterioration in employment status 2 years later.

**Trial registration:**

This observational study is registered under NL43098.008.12: ‘Voorspellers van arbeidsparticipatie bij mensen met relapsing-remitting Multiple Sclerose’. This study is registered at the Dutch CCMO register (https://www.toetsingonline.nl).

**Electronic supplementary material:**

The online version of this article (10.1007/s10072-019-03999-w) contains supplementary material, which is available to authorized users.

## Introduction

Work participation plays an important role in our lives and is often linked to quality of life. Besides income, work participation is known to promote a person’s sense of self-respect and social contacts and provides a feeling of usefulness and satisfaction [1]. Living with a chronic illness can make it challenging to meet the demands of working life. As a result, work participation is often compromised in patients with multiple sclerosis (pwMS) with unemployment rates up to 80% [2]. In those who remain in the workforce, a reduction in hours or work responsibilities, presenteeism and increased time missed from work is often observed [3].

Whether and how pwMS participate in work depends on multiple factors [4, 5]. Physical disability, disease duration, the patient’s age, fatigue, walking problems, cognitive and neuropsychological impairments are linked to difficulties with work participation [4, 6]. Cognitive impairment is present in an estimated 43 to 65% of pwMS and can be present at all stages of the disease [7]. In accordance with the fickle nature of the disease, a wide variety of cognitive deficits can be observed in MS with a great inter-patient variability. In addition, cognitive resources have become more important as the work field over the past decades has changed from an industrial to a post-industrial setting, resulting into a shift from more physically challenging work toward more mentally challenging work [8]. Studies have suggested that cognitive functioning, either self-reported or objectively assessed with a cognitive examination, plays an important role in work participation. Specifically, self-reported cognitive difficulties, global cognitive impairment and lower scores on processing speed/working memory, executive functioning and memory have been linked to worse work outcomes in MS [9–11]. The majority of studies on cognitive functioning and work outcomes are, however, cross-sectional, which makes it impossible to draw conclusions about causality of relationships, and on the predictive value of cognitive functioning regarding work participation in MS. The few longitudinal studies that were conducted found evidence for the predictive value of processing speed/working memory for future employment status [12–14]. Many of the previous studies, including the longitudinal ones, did not examine executive functioning in much detail, while measures of conceptual reasoning, switching and inhibition may be important associates of work functioning [13, 15].

In the current study, we aim to investigate whether baseline cognitive functioning predicts a change in employment status after 2 years, while taking mood, fatigue and disability level into account.

## Materials and methods

### Design and procedure

For this study, pwMS from 16 MS outpatient clinics in the Netherlands were recruited in the context of the MS@Work study, a prospective longitudinal study on work participation in patients with relapsing-remitting MS [5]. The criteria for inclusion were a diagnosis of relapsing-remitting MS [16], patients had to be 18 years and older and currently employed or within 3 years since their last employment. Patients with comorbid psychiatric or neurological disorders, substance abuse, neurological impairment that might interfere with cognitive testing, or inability to speak and/or read Dutch were excluded from the study. A healthy control group was recruited through advertisements on social media and in local newspapers. For the healthy control group, the same inclusion and exclusion criteria were used, except for absence of a chronic disorder.

PwMS underwent neurological and neuropsychological examinations at their outpatient clinics and were asked to fill in online questionnaires yearly for a period of 3 years. The healthy controls underwent a neuropsychological examination at baseline and were asked to fill in the online questionnaires yearly for a period of 3 years. For this study, the baseline and 2-year data were used. The online questionnaires assessed demographic characteristics, work participation, empathy, self-reported cognitive and neuropsychiatric functioning, fatigue and mood.

The MS@Work study was approved by the Medical Ethical Committee Brabant (NL43098.008.12 1307; date of approval: 12-02-2014) and the Board of Directors of the participating MS outpatient clinics. All subjects provided written informed consent. The study was performed in agreement with the Declaration of Helsinki [17].

### Participants

**Fig. 1:**
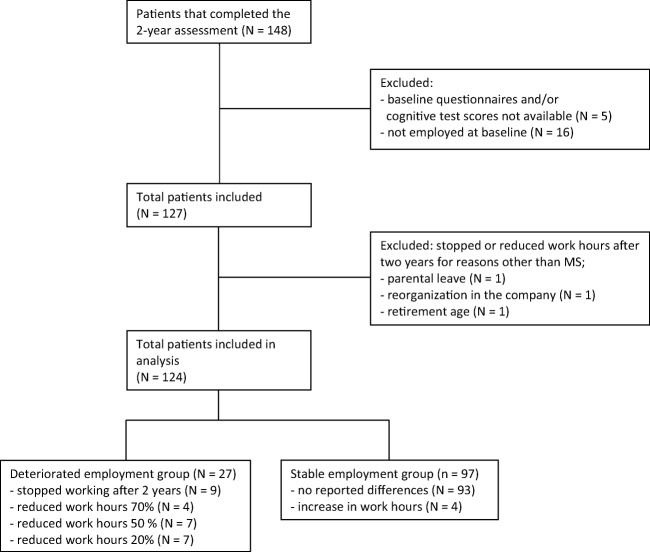
Flow chart of the inclusion of patients with MS

The current study included pwMS who were employed at baseline, completed the baseline assessments and completed questionnaires about their work situation after 2 years (see Fig. 1 for a flow chart of the inclusion of participants). Participants were categorised into a stable employment status (SES) group (*n* = 97) and a deteriorated employment status (DES) group (*n* = 27), based on deteriorations in their work status after 2 years (see measures for a definition). We included 60 healthy controls, matched for age, sex and education to be able to calculate *Z*-scores for cognitive functioning, anxiety, depression and fatigue.

### Measures

#### Neuropsychological examination

The neuropsychological examination included various neuropsychological tests assessing the neurocognitive domains as described in the Diagnostic and Statistical Manual of Mental Disorders-V [18] (i.e. complex attention, learning and memory, language, executive functioning, perceptual motor functioning and social cognition)*.* For an overview of the neuropsychological tests and measures, see Table 1. Most tests are part of the Minimal Assessment of Cognitive Function in MS battery [19]. The Multiple Sclerosis Neuropsychological Screening Questionnaire [20] was used to examine self-reported cognitive and neuropsychiatric functioning. Neuropsychological test scores were transformed into *Z*-scores based on the healthy control group and composite *Z*-scores were calculated per neurocognitive domain.

**Table 1:** Neuropsychological tests and measures used per Diagnostic and Statistical Manual of Mental Disorders-V neurocognitive domain

	Neuropsychological test	Measure
Complex attention	Symbol Digit Modalities Test Trail Making Test Colour Word Interference Test^a^	Total correct Completion time part A Completion time colour naming Completion time reading
Learning and memory	Rey Verbal Learning Test Brief Visuospatial Memory Test-Revised	Total correct immediate recall Total correct delayed recall Total correct immediate recall Total correct delayed recall
Language	Controlled Oral Word Association Test Semantic Category Fluency Test	Total correct Total correct
Executive functioning	Paced Auditory Serial Addition Test Trail Making test Colour Word Interference Test^a^ Design Fluency Test^a^	Total correct on both 3′ and 2′ Completion time part B Contrast score (part B – part A) Completion time inhibition Completion time inhibition/switching Contrast score (inhibition – colour naming Contrast score (inhibition/switching – combined colour naming and word reading)
		Total correct 3 conditions Contrast score (switching – combined full dots and empty dots)
Perceptual motor functioning	Judgement of Line Orientation Test	Total correct
Social cognition	Empathy Quotient^b^	Total score

^a^Delis Kaplan Executive Functioning Systems subtest. ^b^Questionnaire. For a detailed description of the neuropsychological tests and measures, we refer to MS@Work study protocol [5]

#### Employment status

A general questionnaire was used to inquire about demographic characteristics and work participation in both the patients and healthy controls. The general questionnaire was administered at baseline and 2 years after baseline. Participants were regarded to be employed if they were in a paid job or reported to be self-employed. Students, volunteer workers and participants who had reached the retirement age were excluded from analysis.

Participants were divided into SES and DES groups based on deteriorations reported in the 2-year data. A participant was considered to be part of the DES group if they either had stopped working due to MS or had decreased their work hours due to MS by at least 20% based on self-reports. Participants, who reported no differences or had increased their work hours, were included in the SES group.

#### Other clinical measures

The Expanded Disability Status Scale (EDSS) was used to examine physical disability in pwMS [21] and was administered by a neurologist in the outpatient clinic where the patients were being treated. Scores range from 0 (normal neurological exam) to 10 (death due to MS) and increment with steps of 0.5. Scores between 0 and 3.5 represent mild disability, scores between 4.0 and 6.5 represent moderate disability and scores of 7.0 and above represent severe disability [22]. The Hospital Anxiety and Depression Scale [23] was used to examine self-reported symptoms of anxiety and depression. The Modified Fatigue Impact Scale was used to measure the self-reported impact of fatigue on daily functioning [24].

### Statistical analysis

IBM SPSS for Windows (release 23.0) was used for data analysis. *Z*-scores were calculated for the neurocognitive measures, self-reported cognitive functioning, depression, anxiety and fatigue based on the mean and standard deviation (SD) from the healthy control group. *Z*-scores were scaled in such a way that lower scores represent worse functioning and combined into a mean *Z*-score for each neurocognitive domain.

In the first step of analysis, we investigated differences between the SES and DES groups in *Z*-scores per neurocognitive domain and other clinical measures using parametric or non-parametric tests where appropriate.

In the second step of analysis, we conducted a logistic regression analysis, with change in employment status (SES/DES) as dependent variable. As potential predictors, we included the *Z*-scores per neurocognitive domain and the other clinical measures that significantly differed between the DES and SES group as shown in the previous step of the analysis. The level of statistical significance was set at *p* ≤ 0.05.

## Results

### Characteristics of patients with MS

Demographics and disease characteristics of the pwMS and healthy controls are presented in Table 2. Patients and healthy controls did not differ significantly in gender, age, educational level and type of job. The pwMS worked significantly less hours than the healthy controls (*U* = 2246.0, *p* < 0.001). Most pwMS had a white collar job (88.2%), with the majority being employed in office and administrative support (20.5%), the healthcare sector (16.5%) or education sector (11.8%). The pwMS were characterised by mild disability (median 2.0).

**Table 2:** Baseline demographic and disease characteristics of the patients with MS and the healthy control group

	Patients with MS					Healthy control group				
	*N*	%	Mean (SD)	Median (IQR)	Min–max	*N*	%	Mean (SD)	Median (IQR)	Min-max
Gender (% female)	124	82.3%				60	80%			
Age	124		42.3 (9.0)	43.0 (13.0)	21–63	60		41.9 (10.3)	44.0 (15.0)	20–64
Educational level	124					60				
Low	21	16.9%				3	5.0%			
Medium	47	37.9%				27	45.0%			
High	56	45.2%				30	50.0%			
Number of work hours per week	124		26.2 (11.0)	24.0 (18.0)	3–55	60		33.4 (8.3)	36.0 (12.0)	12–50
Type of work (%white collar)	124	87.9%				60	81.7%			
Expanded Disability Status Scale	115^a^		2.1 (1.3)	2.0 (1.0)	0–6	n.a.				
Medication (% using immunomodulatory treatment)	124	79.8%				n.a.				
Disease duration (in years)	124		7.6 (6.2)	6.0 (7.8)	0–28	n.a.				

Education was divided into three levels: low education (up to finishing low-level secondary school), middle education (finishing secondary school at a medium level) or high education (finished secondary school at the highest level and/or obtained a college or university degree). ^a^Neurological data was not available for 9 patients with MS; *n.a*., not applicable

In Table 3, the mean *Z*-scores for the neurocognitive domains, self-reported cognitive functioning, anxiety, depression and fatigue are presented. The neurocognitive domains where patients most frequently scored below average (≤ 2SD as compared with healthy controls) are complex attention (8.9%), followed by perceptual motor functioning (5.7%) and learning and memory (3.2%). A total of 20 pwMS (16.1%) scored below average (≤ 2SD) on at least one neurocognitive domain. A total of 8.9% and 10.5% of the pwMS reported scores indicative of major depression and generalised anxiety, and 26.6% reported scores indicative of MS-related fatigue (based on ≤ 2SD).

**Table 3:** *Z*-scores for each DMS-V neurocognitive domain, self-reported cognitive functioning, anxiety, depression and fatigue for patients with MS

	*N*	Mean *Z*-score	Min–max	*N* with *Z*-score ≤ − 2	% with *Z*-scores ≤ − 2
Complex attention	124	− 0.37	− 4.68–2.12	11	8.9%
Learning and memory	124	− 0.31	− 3.71–1.38	4	3.2%
Language	124	− 0.52	− 2.24–2.40	3	2.4%
Executive functioning	119^a^	− 0.04	− 1.38–1.47	0	0.0%
Perceptual motor functioning	123^b^	− 0.24	− 4.17–1.00	7	5.7%
Social cognition	124	− 0.11	− 2.72–1.93	2	1.6%
Self-reported cognitive functioning	124	− 0.44	− 4.77–2.37	14	11.3%
Self-reported anxiety	124	− 0.55	− 4.72–1.61	13	10.5%
Self-reported depression	124	− 0.63	− 5.61–0.65	11	8.9%
Self-reported fatigue	124	− 1.16	− 4.29–1.54	33	26.6%

^a^Data not complete due to technical issues (*N* = 3) or wrong administration (*N* = 2) of the Paced Auditory Serial Addition Test and Design Fluency. ^b^Data missing due to unknown reason

### Baseline comparisons between the SES and DES groups

Baseline comparisons between the SES and DES groups in demographic characteristics, cognitive functioning and clinical characteristics are presented in Table 4. In 27 cases, patient’s employment deteriorated (DES; 22%). Of the 97 that were in the SES group (78%), four patients reported an increase in work hours. The SES and DES groups did not differ in gender, age, educational level, work hours, type of work, the use of immunomodulatory treatment, disease duration, learning and memory, language, perceptual-motor functioning, social cognition and anxiety at baseline. The DES group showed more physical disability, lower complex attention and executive functioning, more self-reported cognitive problems, more symptoms of depression, and more fatigue than the SES group at baseline.

**Table 4:** Baseline comparisons in demographic characteristics, cognitive functioning, anxiety, depression and fatigue between the stable and deteriorated employment groups

	Stable employment status			Deteriorated employment status			Test statistics	
	*N*	%, mean (SD), median (IQR)	Min–max	*N*	%, mean (SD), median (IQR)	Min–max	*X*^2^, *T*-statistic, *U*-statistic	*p* value
Demographic characteristics								
Gender (% female)	97	80.4%		27	88.9%		*X*^2^ = 1.0	*p* = 0.31
Age, mean (SD)	97	42.2 (9.2)	21–63	27	42.6 (8.6)	29–58	*T* = − 0.2	*p* = 0.84
Educational level	97			27			*X*^2^ = 1.0	*p* = 0.60
Low (%)	16	16.5%		5	18.5%			
Medium (%)	39	40.2%		8	29.6%			
High (%)	42	43.3%		14	51.9%			
Number of work hours, median (IQR)	97	25.0 (17.5)	3–55	27	24.0 (20.0)	8–40	*U* = 1224.0	*p* = 0.60
Type of work (% white collar)	97	85.6%		27	96.3%		*X*^2^ = 2.3	*p* = 0.13
Expanded Disability Status Scale, median (IQR)	91	2.0 (1.0)	0–6	24	3.0 (2.0)	0–6	*U* = 557.0	*p* < 0.001*
Disease duration (in years), median (IQR)	97	5.0 (8.5)	0–28	27	7.0 (7.0)	1–26	*U* = 1170.5	*p* = 0.40
Medication (% using immunomodulatory treatment)	97	79.4%		27	81.5%		*X*^2^ = 0.1	*p* = 0.81
Cognitive functioning								
Complex attention, median (IQR)	97	− 0.20 (1.05)	− 4.68–2.12	27	− 0.53 (1.49)	− 4.49–0.86	*U* = 989.0	*p* = 0.05*
Learning and memory, mean (SD)	97	− 0.29 (0.84)	− 3.42–1.38	27	− 0.38 (1.15)	− 3.71–1.04	*T* = 0.4	*p* = 0.67
Language, mean (SD)	97	− 0.48 (0.74)	− 2.02–2.40	27	− 0.65 (0.86)	− 2.24–1.37	*T* = 1.0	*p* = 0.33
Executive functioning, mean (SD)	92	0.05 (0.55)	− 1.29–1.47	27	− 0.35 (0.55)	− 1.38–0.71	*T* = 3.3	*p* = 0.001*
Perceptual motor functioning, median (IQR)	97	− 0.09 (1.09)	− 3.63–1.00	26	− 0.50 (1.36)	− 4.17–1.00	*U* = 1121.5	*p* = 0.39
Social cognition, mean (SD)	97	− 0.16 (0.81)	− 2.72–1.93	27	0.05 (0.96)	− 1.79–1.65	*T* = − 1.1	*p* = 0.26
Self-reported cognitive functioning, mean (SD)	97	− 0.29 (1.35)	− 4.77–2.37	27	− 0.98 (1.20)	− 3.20–1.52	*T* = 2.4	*p* = 0.02*
Other clinical measures								
Self-reported anxiety, median (IQR)	97	− 0.37 (1.58)	− 4.32–1.61	27	− 0.37 (1.58)	− 4.72–1.21	*U* = 1260.5	*p* = 0.77
Self-reported depression, median (IQR)	97	− 0.19 (1.25)	− 3.94–0.65	27	− 1.02 (2.09)	− 5.61–0.65	*U* = 935.5	*p* = 0.02*
Self-reported fatigue, median (IQR)	97	− 1.18 (1.48)	− 4.14–1.54	27	− 1.57 (1.71)	− 4.29–0.60	*U* = 862.5	*p* = 0.007*

*p values of ≤ 0.05 are considered significant

### Predictors of changes in employment status after 2 years

Results of the logistic regression analysis are presented in Table 5. Lower executive functioning and more physical disability were retained as independent predictors of DES. All other variables did not independently contribute to the prediction.

**Table 5:** Logistic regression model of stable or deteriorated employment after 2 years

Included	*B*	S.E.	OR	[95% CI OR]	*p* value
Constant	− 3.70	0.78			*p* ≤ 0.001*
Complex attention	− 0.14	0.27	0.87	[0.51–1.47]	*p* = 0.60
Executive functioning	− 1.20	0.55	0.30	[0.10–0.88]	*p* = 0.03*
Self-reported cognitive functioning	− 0.18	0.32	0.84	[0.45–1.57]	*p* = 0.58
Self-reported depression	0.36	0.29	1.43	[0.81–2.51]	*p* = 0.22
Self-reported fatigue	− 0.58	0.38	0.56	[0.27–1.18]	*p* = 0.13
Expanded Disability Status Scale	0.64	0.25	1.90	[1.16–3.10]	*p* = 0.01*

Model: *R*^2^ = 0.22 (Cox & Snell), *R*^2^ = 0.33 (Nagelkerke), *Χ*^2^(6) = 26.9 *p* ≤ 0.001. *p values of ≤ 0.05 are considered significant. The model included = 110 patients with MS due to missing data on physical disability and executive functioning

## Discussion

This longitudinal study shows that pwMS who deteriorated in employment status after 2 years due to MS showed lower executive functioning, more self-reported cognitive problems, more symptoms of depression, higher fatigue and higher physical disability (i.e. higher EDSS scores) at baseline. These findings are in accordance with factors that have previously been linked to worse employment outcomes in cross-sectional studies [6]. Our logistic regression model retained more physical disability and lower executive functioning as independent baseline predictors of a deterioration in employment status over a 2-year period. All other variables did not significantly predict DES 2 years after baseline. Both physical disability and executive functioning have previously been linked with work participation in multiple (mostly) cross-sectional studies [6, 9]. Although the level of physical disability in our group of pwMS can be considered mild, it seems that more physical disability is an important risk factor for less favourable employment outcomes over time.

None of the pwMS in this study showed below average (< 2SD) executive functioning at baseline. Still, lower executive functioning was found to be predictive of DES. This is not surprising, because executive functioning is thought to be crucial for successful work participation, as most job demands include executive abilities such as problem solving, planning and reasoning [25].

Comparing our results with the few other longitudinal studies, Morrow et al. (2010) found that both baseline and a decline in processing speed/working memory (Symbol Digit Modalities Test) and verbal memory predicted a deterioration in vocational status 3 years later [12]. Ruet et al. (2013) found that lower baseline processing speed/working memory (Symbol Digit Modalities Test and Paced Auditory Serial Addition Test), more physical disability and higher age were significantly associated with a decreased employment status after 7 years [13]. In the register-based cohort study by Kavaliunas et al. (2019), a relation between baseline lower processing speed (Symbol Digit Modalities Test) and work disability (sickness absence and disability pensioned) at 1 and 3 years after follow-up was found [14].

All these longitudinal studies found evidence for the influence of processing speed/working memory on future employment status, but did not comprehensively evaluate executive functioning.

In our study, the neurocognitive domain of executive functioning reflects the combined performance on the Paced Auditory Serial Addition Test and complex conditions of the Trail Making Test, Colour Word Interference Test and Design Fluency. Upon further inspection, we found that specifically the scores on the Paced Auditory Serial Addition Test and Colour Word Interference Test differed between the SES and DES groups (see online supplementary material). The Paced Auditory Serial Addition Test has been previously linked to employment outcomes in pwMS [19, 26] and is regarded as one of the most sensitive measures for employment outcomes, together with the Symbol Digit Modalities Test [11–13, 27]. The Colour Word Interference Test measures both inhibition and cognitive flexibility [28] and may also be influenced by a processing speed component. In the little research that has used the Colour Word Interference Test as a measure of executive functioning in combination with employment status, no associations were found [29, 30]. However, the Colour Word Interference Test may provide a measure of inhibition and cognitive flexibility (either combined with processing speed) that is representative of the ability to overcome challenges faced by individuals with MS in the workplace.

Although we observed group differences in self-reported cognitive functioning, fatigue and depression between the SES and DES groups, these variables were not retained as independent predictors of DES after 2 years. Self-reported cognitive functioning as measured with the Multiple Sclerosis Neuropsychological Screening Questionnaire was previously found to be related with employment status [31] and negative work events [32]. Furthermore, previous research shows strong evidence that both fatigue and depression are associated with work outcomes [6]. A possible explanation for the lack of baseline predictive power is that these variables are subject to time-dependent changes. It is known that depression as well as anxiety can change over time in pwMS [33]. In line, it seems intuitive that fatigue scores fluctuate over time influenced by primary and secondary mechanisms (e.g. sleep disorder, depression, iatrogenic mechanisms) [34]. It would be interesting to further investigate change scores of fatigue, depression, anxiety and self-reported cognitive functioning over time in relation to changes in employment outcomes.

Even though physical disability and executive functioning are predictors of DES, together they were only able to explain a small part of the variance in DES. Work participation in MS is a multifactorial problem and cannot solely be explained by the measured variables. It is known that personal- and work-related factors are related to work participation. For example, the willingness of companies to facilitate suitable adaptations like flexible work schedule or accommodations to the workload may be as important [35]. To date, the amount of studies investigating these (and other) personal- and work-related factors is limited and it might be of great interest to investigate these factors more extensively.

### Strengths and limitations

Strengths of the current study include its longitudinal character, the use of a matched control group and the use of a comprehensive neuropsychological assessment. The study sample was characterised by working patients with relapsing-remitting MS, mild physical disability and a low prevalence of patients showing below average performance on the six neurocognitive domains (0–8.9%), and thus its findings may probably not be generalisable to the entire MS population. Furthermore, with the use of questionnaires, our self-reported work measure may be subject to a recall bias.

In our outcome measure, we did not include presenteeism or qualitative deteriorations in employment, like loss of responsibilities or other changes of function due to MS. PwMS that worked in a less skilled job for the same number of work hours 2 years after baseline, were not considered to be in the deteriorated group. In this regard, it is possible that pwMS in the stable group did qualitatively deteriorate in employment status and thus, the effects found could possibly be an underestimation of the true effect.

In the current study, we have taken into account various influential factors like mood, fatigue and disability level. Another possible influential factor, that we did not take into account, is the use of immunomodulatory treatment and switches in treatment over time. Most patients in our sample use disease-modifying drugs, and some of these drugs have been reported to affect cognitive [36] and work functioning [37]. In future studies, it might be interesting to also take into account the potential effects of (changes in) disease-modifying drugs on cognitive functioning and work participation over time. In future research, it would be relevant to investigate changes in cognitive functioning in relation to work participation over time. The only two prospective, longitudinal studies that used a cognitive test battery found that changes in employment status after 3 and 7 years were predicted by both baseline cognitive performance and cognitive deterioration during those years [12, 13]. In future research, the relation between work participation and a possible decrease in cognitive functioning and other clinical-, personal- and work-related factors over time should be investigated. As data collection of the MS@Work study is still ongoing, we plan to focus on this issue in the future.

Although we used a matched control group to calculate standardised scores for the neurocognitive measures, it would be interesting to use meaningful and work-specific reference criteria, such as minimal cognitive ability required to successfully perform a certain work task. This is in line with the model of workload (mental and physical task load) and work capacity (ability to execute a task) [38]. Similar work-related reference values have been established for a functional capacity evaluation of physical functioning [39]. However, references are currently unavailable for cognitive functioning in relation to work, which limits meaningful interpretation of neuropsychological functioning in terms of work capacity, and imposes a relevant venue for future research.

## Conclusion

The current study revealed that lower executive functioning and more physical disability are moderately predictive of a deterioration in employment status after 2 years due to MS. Mood and fatigue were not retained as independent predictors of a deterioration in employment status. We should keep in mind that work participation in pwMS is a multifactorial problem. Besides disease-related factors, it might be of great interest to investigate personal- and work-related factors more extensively.

## Electronic supplementary material

ESM 1 (DOC 67 kb)
