# Supplementary material for: Cognitive functioning as a predictor of employment status in relapsing-remitting multiple sclerosis: a 2-year longitudinal study
Source: Neurol Sci. 2019 Jul 19;40(12):2555–64. doi: 10.1007/s10072-019-03999-w (PMC6848242; doi:10.1007/s10072-019-03999-w)
Supplement: Supplementary file 1 — (DOC 67 kb) [file 10072_2019_3999_MOESM1_ESM.doc]

# Online Supplementary Material

Table S1: Descriptive table of Z-scores test comparisons between the stable and deteriorated employment groups.

|  | Stable employment  group | | | Deteriorated employment  group | | | Test statistics | |
| --- | --- | --- | --- | --- | --- | --- | --- | --- |
|  | N | %,  mean (SD),  median (IQR) | Min-Max | N | %,  mean (SD),  median (IQR) | Min-Max | T-statistic,  U-statistic | P value |
| **Complex attention** |  |  |  |  |  |  |  |  |
| SDMT, *median (IQR)* | 97 | -0.22 (1.18) | -2.72 – 4.26 | 27 | -1.01 (1.97) | -3.11 - 0.84 | U = 801.0 | P = 0.002* |
| TMT part A, *median (IQR)* | 97 | -0.02 (1.59) | -4.24 – 1.44 | 27 | -0.16 (1.72) | -3.98 – 1.44 | U = 1189.0 | P = 0.47 |
| CWIT colour naming (1), *median (IQR)* | 97 | 0.05 (1.50) | -9.36 – 2.02 | 27 | 0.05 (2.21) | -6.93 - 0.94 | U = 1084.0 | P = 0.17 |
| CWIT time reading (2), *median (IQR)* | 97 | 0.06 (1.29) | -9.91 – 1.81 | 27 | -0.11 (1.78) | -8.41 – 1.42 | U = 1153.5 | P = 0.34 |
| **Learning and memory** |  |  |  |  |  |  |  |  |
| 15WT immediate recall , *mean (SD)* | 97 | -0.55 (1.12) | -3.76 – 1.75 | 27 | -0.58 (1.16) | -2.66 – 1.50 | T = 0.1 | P =0.90 |
| 15WT delayed recall , *median (IQR)* | 97 | -0.61 (1.79) | -3.74 – 1.64 | 27 | -0.61 (2.24) | -3.29 – 1.19 | U = 1300.5 | P = 0.96 |
| BVMT-R immediate recall, *median (IQR)* | 97 | 0.15 (1.40) | -3.06 – 1.75 | 27 | 0.35 (1.60) | -4.06 – 1.55 | U = 1204.0 | p = 0.52 |
| BVMT-R delayed recall, *median (IQR)* | 97 | 0.21 (1.39) | -4.65 - 0.90 | 27 | 0.21 (1.39) | -5.34 – 0.90 | U = 1243.0 | p = 0.68 |
| **Language** |  |  |  |  |  |  |  |  |
| COWAT total correct, *mean (SD)* | 97 | -0.40 (0.87) | -2.09 – 2.54 | 27 | -0.54 (0.80) | -1.74 – 1.56 | T = 0.7 | P = 0.46 |
| SCF total correct, *mean (SD)* | 97 | -0.57 (0.80) | -2.31 – 2.27 | 27 | -0.76 (1.10) | -2.75 – 1.72 | T = 1.0 | P = 0.32 |
| **Executive functioning** |  |  |  |  |  |  |  |  |
| PASAT 3’ + PASAT 2’ *mean (SD)* | 94 | -0.02 (0.88) | -2.23 – 1.61 | 27 | -0.61 (0.85) | -2.40 – 1.55 | T = 3.1 | P = 0.003* |
| TMT part B, *median (IQR)* | 97 | 0.14 (1.02) | -4.44 – 1.04 | 27 | -0.35 (1.30) | -2.40 – 1.13 | U = 1008.5 | P = 0.07 |
| TMT contrast score (B-A), *median (IQR)* | 97 | 0.27 (0.81) | -5.41 – 1.87 | 27 | -0.19 (1.09) | -2.45 – 1.09 | U = 1000.0 | P = 0.06 |
| CWIT inhibition (3), *median (IQR)* | 97 | 0.35 (0.96) | -2.98 – 2.59 | 27 | -0.31) 1.10) | -4.32 – 1.31 | U = 843.0 | P = 0.005* |
| CWIT inhibition/switching (4), *median (IQR)* | 97 | 0.04 (0.88) | -2.55 – 1.57 | 27 | -0.49 (1.62) | -3.31 – 1.05 | U = 893.0 | P = 0.01* |
| CWIT contrast (inhibition – colour naming), *median (IQR)* | 97 | 0.27 (1.06) | -3.66 – 7.65 | 27 | 0.01 (1.41) | -3.32 – 1.55 | U = 978.5 | P = 0.05* |
| CWIT contrast (inhibition/switching –  combined colour naming + word reading), *mean (SD)* | 97 | 0.10 (0.79) | -2.49 – 2.24 | 27 | -0.42 (1.18) | -2.89 – 2.15 | T = 2.18 | P = 0.04* |
| DF total correct, *median (IQR)* | 95 | 0.04 (1.54) | -2.59 – 2.51 | 27 | -0.27 (1.08) | -1.81 – 1.43 | U = 1053.5 | p = 0.16 |
| DF contrast (switching –  combined full dots + empty dots), *mean (SD)* | 95 | 0.03 (0.92) | -2.49 – 3.25 | 27 | 0.12 (0.88) | -1.62 – 1.51 | T = -0.4 | p = 0.66 |
| **Perceptual motor functioning** |  |  |  |  |  |  |  |  |
| JOLOT total correct, *median (IQR)* | 97 | -0.09 (1.09) | -3.63 – 1.00 | 26 | -0.50 (2.04) | -4.17 – 1.00 | U = 3133.5 | P = 0.06 |
| **Social cognition** |  |  |  |  |  |  |  |  |
| EQ, *mean (SD)* | 97 | -0.16 (0.81) | -2.72 – 1.93 | 27 | 0.05 (0.95) | -1.79 – 1.65 | T = -1.1 | P = 0.26 |
| **Self-reported cognitive functioning** |  |  |  |  |  |  |  |  |
| MSNQ – patient, *mean (SD)* | 97 | -0.29 (1.35) | -4.77 – 2.37 | 27 | -0.98 (1.20) | -3.20 – 1.52 | T = 2.4 | P =0.02* |
| **Mood** |  |  |  |  |  |  |  |  |
| HADS depression, *median (IQR)* | 97 | -0.19 (1.25) | -3.94 - 0.65 | 27 | -1.02 (2.09) | -5.61 - 0.65 | U = 935.5 | P = 0.02* |
| HADS anxiety, *median (IQR)* | 97 | -0.37 (1.58) | -4.32 – 1.61 | 27 | -0.37 (1.58) | -4.72 – 1.21 | U = 1260.5 | P =0.77 |
| **Fatigue** |  |  |  |  |  |  |  |  |
| MFIS, *median (IQR)* | 97 | -1.18 (1.48) | -4.14 – 1.54 | 27 | -1.58 (1.71) | -4.29 - 0.60 | U = 862.5 | P = 0.007* |

Note; * P values of ≤.05 are considered significant. SD: Standard Deviation, IQR: Inter Quartile Range. Tests used; SDMT: Symbol Digit Modalities Test, TMT: Trail Making Test, CWIT: Colour Word Interference Test, 15WT, Groningen 15 Word Learning Task, BVMT-R: Brief Visuospatial Memory Test-Revised, COWAT: Controlled Oral Word Association Test, SCF: Semantic Category Fluency Test, PASAT: Paced Auditory Serial Addition Test, DF: Design Fluency test, JOLOT: Judgement of Line Orientation, EQ: Empathy Quotient, MSNQ: Multiple Sclerosis Neuropsychological screening Questionnaire, HADS: Hospital Anxiety and Depression Scale, MFIS: Modified Fatigue Impact Scale.
